# *Leishmania major* infection in a dog with cutaneous manifestations

**DOI:** 10.1186/s13071-016-1541-2

**Published:** 2016-05-10

**Authors:** Gad Baneth, Yaarit Nachum-Biala, Maytal Shabat Simon, Ori Brenner, Sarit Gaier, Alicia Rojas, Daniel Yasur-Landau

**Affiliations:** School of Veterinary Medicine, Hebrew University, P.O. Box 12, Rehovot, 76100 Israel; Tel Aviv University, Ramat Aviv, Tel Aviv, Israel; Department of Veterinary Resources, Weizmann Institute of Science, Rehovot, Israel; Veterinary Center, Hadera, Israel

**Keywords:** *Leishmania major*, Cutaneous leishmaniasis, Canine, Israel, Allopurinol, Molecularly-confirmed, *Leishmania major* treatment

## Abstract

**Background:**

*Leishmania major* is a main cause of cutaneous leishmaniasis in humans in an area that stretches from India through Central Asia, the Middle East, to North and West Africa. In Israel, it is a common infection of humans with rodents as the reservoir hosts and *Phlebotomus papatasi* as its sand fly vector.

**Findings:**

A 6 months old spayed female mixed breed dog was referred to the Hebrew University Veterinary Teaching Hospital with a large ulcerative dermal lesion on the muzzle, and lesions in the foot pads and left hind leg. Histopathology of a skin biopsy found chronic lymphohistiocytic dermatitis with the presence of *Leishmania* spp. amastigotes in the muzzle. Physical examination indicated that the dog was overall in a good clinical condition and the main findings were the skin lesions and enlarged prescapular lymph nodes. Complete blood count and serum biochemistry profile were within reference ranges. Serology by ELISA was positive for *Leishmania* spp. and PCR of the prescapular lymph node was positive by an ITS1 region PCR-high resolution melt analysis. However, the melt curve and subsequent DNA sequencing indicated that infection was caused by *L. major* and not *L. infantum*, which is the main causative agent of canine leishmaniosis in the Mediterranean region. DNA was extracted from the paraffin embedded muzzle biopsy and PCR with sequencing also indicated *L. major*. The dog’s young age and the absence of hyperglobulinemia and anemia were not typical of *L. infantum* infection. The dog was treated with allopurinol and the skin lesions improved and later disappeared when the dog was re-evaluated.

**Conclusions:**

This is the first molecularly-confirmed case of *L. major* infection in a dog. Two previous reports of *L. major* in dogs originated from Saudi-Arabia and Egypt in 1985 and 1987 were confirmed by enzymatic biochemical techniques. Serology for *L. infantum* was positive probably due to the well documented serological cross-reactivity between *Leishmania* spp. Although dogs and wild carnivores are not considered main reservoirs for *L. major*, the possibility of clinical canine disease and their potential as secondary hosts should be investigated in areas endemic for human *L. major* infection.

## Background

*Leishmania major* causes human cutaneous leishmaniasis in Asia and Africa. In the Middle East and Israel, it is a common cause of human infection with rodents as reservoir hosts and *Phlebotomus papatasi* as its sand fly vector.

## Clinical case

**Fig. 1:**
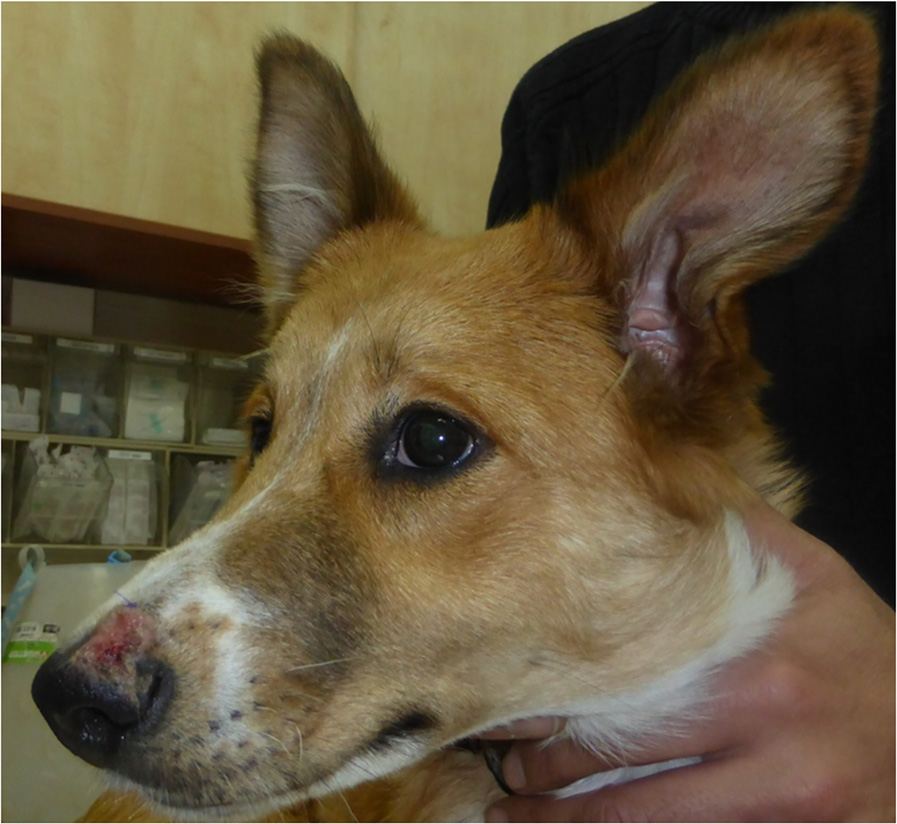
Cutaneous *Leishmania major* in the dog. Dog showing cutaneous lesion caused by *Leishmania major* on the muzzle before treatment

**Fig. 2:**
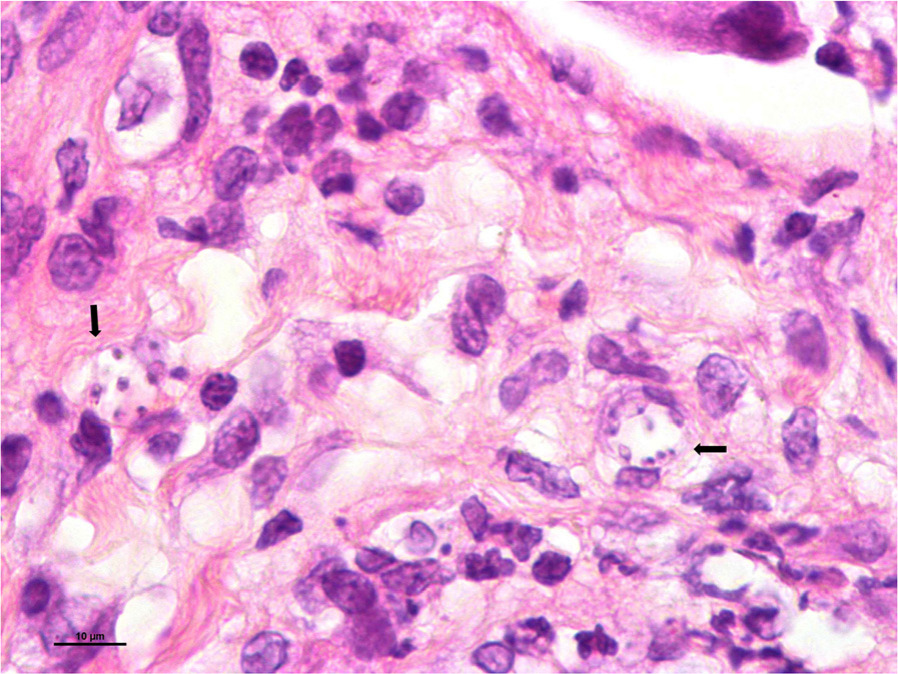
Histological section, muzzle skin lesion. Arrows point to groups of round to oval *Leishmania* spp. amastigotes, approximately 1 to 2 μm in size, with small basophilic nuclei located in the cytoplasm of macrophages. Hematoxylin & eosin stain. *Scale-bar*: 10 μM

A 6-month-old mixed-breed female dog from Hadera, on the coastal plain of Israel, was presented in February 2015 to a local veterinary clinic with skin wounds over the muzzle and foot pads and an ulcerative dermal lesion over the left tarsal joint. The dog was adopted at the age of four months from an animal shelter in Tel Aviv and its birthplace was unknown. The lesion on the muzzle was 3 × 4 centimeters in size and composed of a deep skin ulcer with bloody discharge surrounded by an elevated granulating rim (Fig. 1). A full thickness punch biopsy of the muzzle skin lesion was taken under general anesthesia when the dog was neutered and submitted for histological evaluation. Microscopically, the skin showed chronic lymphohistiocytic and granulomatous dermatitis. There was a severe multifocal and coalescing interstitial and perifollicular histiocytic and lymphocytic dermal infiltration with plasma cells and a small number of neutrophils. Macrophages had an expanded vacuolated cytoplasm and in some, there were groups of round to oval organisms, approximately 1 to 2 μm in size, with small basophilic nuclei interpreted as *Leishmania* spp. amastigotes (Fig. 2). Following the presumptive diagnosis of leishmaniosis, serology for *Leishmania infantum* was submitted to the Hebrew University School of Veterinary Medicine (HUSVM) and performed by ELISA as previously described [1]. ELISA serology with *L. infantum* antigen was low positive with an optical density (O.D.) of 0.79 (cut-off 0.6 O.D.). The dog was then referred to the HUSVM for further diagnosis.

**Fig. 3:**
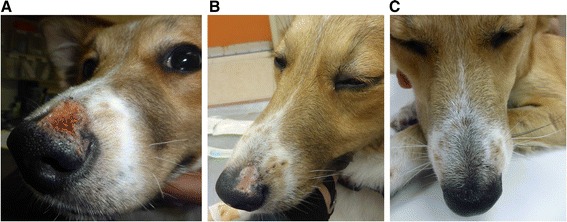
Progression of cutanous muzzle lesion healing during allopurinol treatment. **a** Lesion before treatment. **b** Lesion after 3 weeks of treatment. **c** Disappearance of lesion after 19 weeks of treatment

On physical examination, the dog had a normal body temperature and good general body condition. The left prescapular lymph node was moderately enlarged and ulcerative skin lesions were found on the muzzle (Figs. 1, 3a) and over the left tarsus, and the right front and hind footpads. A complete blood count (CBC), serum biochemistry panel and urinalysis were taken, as well as blood and lymph-node aspirate for PCR using the ITS1-PCR-high resolution melt (HRM) analysis [2].

Blood count, serum biochemistry and urinalysis results were within normal limits with no hyperglobulinemia or hypoalbuminemia typical of canine *L. infantum* infection [3]. Blood was negative by PCR; however, the prescapular lymph node was positive and yielded a DNA sequence that was 100 % identical to *Leishmania major* (GenBank KM052753) as found by BLAST analysis (http://www.ncbi.nlm.nih.gov/BLAST). Following this, the muzzle biopsy taken earlier by the referring veterinarian was received from the pathology service, sample was deparaffinized and DNA was extracted from the tissue using the QIAamp DNA FFPE tissue kit (QIAgen, Valencia, CA, USA) according to the manufacturer’s instructions. PCR from the muzzle biopsy performed by the ITS1-HRM-PCR was also positive for *L. major* and confirmed by DNA sequencing as 100 % identical to the same GenBank accession as the dog’s lymph node sequence (KM052753). The dog was diagnosed as having *L. major* infection and long-term treatment with allopurinol at 10 mg/kg every 12 h was initiated as recommended for dogs infected with *L. infantum* [3]. The owners were also advised to fit a topical insecticide sand fly repelling collar on the dog.

On a follow-up visit twenty one days after the initiation of treatment, the dog’s skin lesions had improved and the muzzle lesion had shrunk and showed progressive healing (Fig. 3b), but the left prescapular lymph node was still mildly enlarged. The CBC showed a mild leukocytosis (17.0 × 10^9^ leukocytes/l; reference 5.2–13.9) with neutrophilia (12.0 × 10^9^ neutrophils/l; reference 3.9–8.0) and serum biochemistry indicated a mild hypoalbuminemia (28 g/l; reference 30–44) with normal globulin levels (30 g/l; reference 23–53). ITS1-HRM-PCR of tissue aspirates from the two prescapular lymph nodes was positive and compatible with *L. major* after sequencing, and the blood was PCR-negative again as well as PCR of conjunctival swabs which were negative. Repeat serology was positive (1.3 O.D.). Parasite culture from a pre-scapular lymph node aspirate seeded in NNN slants overlaid with Schneider’s *Drosophila* medium as previously described was positive [1]. Cultures grew *Leishmaia* promastigotes which were characterized by ITS1-HRM-PCR and DNA sequencing as *L. major*. Unfortunately parasites in these cultures did not continue to propagate and eventually growth was halted and parasites could not be maintained longer.

The dog was brought for a third follow-up 59 days after treatment initiation at which the muzzle skin lesion had almost disappeared, there were residual scars on the foot pads and the prescapular lymph nodes were mildly enlarged. ELISA serology at this time was considered negative for *L. infantum*-infected dogs (0.29 O.D.) and PCR from the pre-scapular lymph node was positive for *L. major*, while blood PCR was again negative. A fourth and final follow-up visit took place 19 weeks after treatment initiation while the dog continued to receive the allopurinol treatment. The muzzle (Fig. 3c) and footpad skin lesions were not evident any longer and the pre-scapular lymph nodes were small and not readily available for sampling by needle aspiration. The owners reported that the dog was active and in good health. ELISA serology was negative with an even lower O.D. (0.12) than found in the previous visit and blood PCR was negative. The dog continued to be treated with allopurinol at the same dose for one year and follow-up visits to the attending veterinarian were recommended every 6 months.

**Fig. 4:**
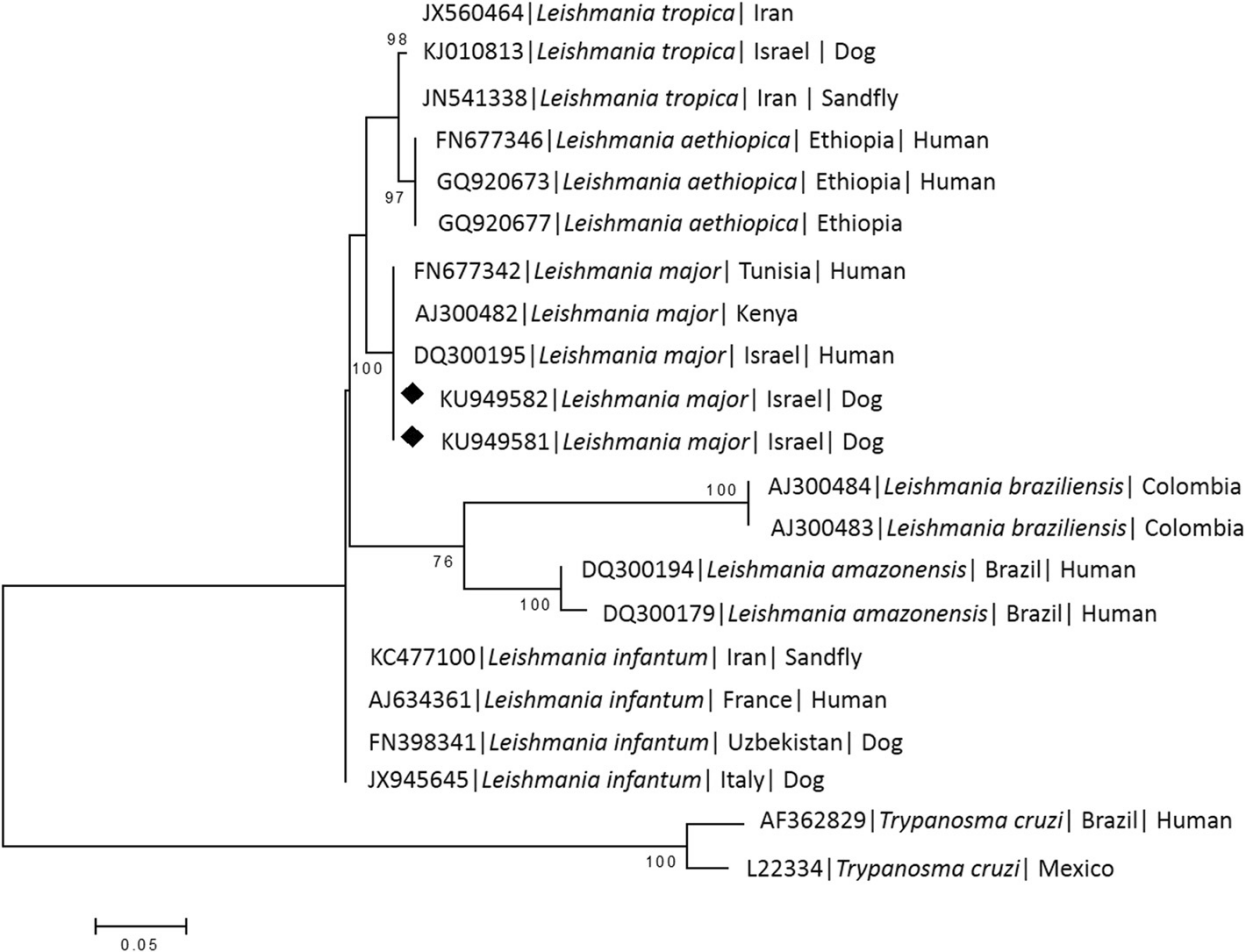
A maximum likelihood phylogram comparing 391-bp DNA sequences of the ITS1/5.8S/ITS2 locus from the dog included in the study to sequences from other *Leishmania major* strains and of other *Leishmania* spp. sequences deposited in GenBank. New sequences derived from this study are marked with black diamond squares. The GenBank accession numbers, species of infected host (when known) and country of origin are included for each sequence. The Tamura-3-Parameter model was used in the construction of this phylogram and bootstrap values higher than 70 % are indicated

Additional 400 bp ITS1 fragments were amplified from the parasite promastigote culture and from the muzzle skin biopsy using primers ITS1F and ITS2R4 [4] as previously described [5] in order to further characterize the *L. major* strain infecting the dog. The DNA sequences of these amplified ITS1 loci from the two sampled tissues were found identical and submitted to GenBank (KU949581; KU949582). A phylogram of these amplified *L.* major ITS1 sequences was constructed to compare them to other *L. major* strains and additional *Leishmania* spp. present in GenBank. Sequences were analyzed using the MEGA version 6.0 software [6] (http://www.megasoftware.net) and a phylogram was constructed by the Maximum likelihood algorithm with the Tamura-3-parameter model [7]. Bootstrap replicates were performed to estimate the node reliability, and values were obtained from 1000 randomly selected samples of the aligned sequence data (Fig. 4). The phylogram indicated that the dog’s *L. major* sequences clustered together with other *L. major* strains from humans in Israel and other countries, separately from *L. infantum* and *L. tropica* strains from dogs and humans, and also away from *L. aethiopica*, *L. amazonensis* and *L. braziliensis*.

## Discussion

*Leishmania major* is a main cause of cutaneous leishmaniasis in humans in an area that stretches from India through Central Asia, the Middle East, to North and West Africa. In Israel, it is a common infection of humans with different species of rodents as the reservoir hosts and *Phlebotomus papatasi* as its sand fly vector [8]. A resurgence of human cutaneous leishmaniasis has been reported in Israel during 2001–2012, with increased infection of both *L. major* and *L. tropica* with an annual incidence of 4.4 cases per 100,000 inhabitants in 2012 [9]. A sevenfold increase in the laboratory-confirmed cutaneous leishmaniasis cases was observed from 2007 to 2013 in southern Israel where *L. major* is the main cause of this disease [10].

Different *Leishmania* spp. have been reported to infect dogs in a variety of regions in the world [11], however, to our best knowledge, only two previous reports of natural canine *L. major* infection have been published. These descriptions which originated from Saudi-Arabia and Egypt in 1985 and 1987, respectively, were confirmed for infection with *L. major* by enzymatic biochemical techniques [12–15]. While *L. major* was apparently isolated from the scrotum and an ear ulcer of Saudi-Arabian dogs [12, 14], isolation was made from the spleen of an emaciated dog with diarrhea and from the blood of a lethargic dog with mild alopecia, both in Egypt [15]. No additional clinical information is available on these dogs, no serology was carried out and confirmation by molecular biology techniques was not done as it was unavailable at that time. Furthermore, no treatment of these dogs has been reported. Our report is thus the first description of clinical *L. major* infection in a dog which was confirmed by molecular methods and the first to follow the treatment of disease and report its progression.

The affected dog described here was young, had only cutaneous manifestations of disease and did not develop the common CBC and serum biochemistry abnormalities of dogs with *L. infantum* infection such as anemia and hyperglobulinemia [3]. Although both skin lesions and superficial lymph nodes were positive for *L. major* by PCR and culture, and the dog was seropositive for *Leishmania* spp. by ELISA, PCR of the blood was consistently negative and no systemic manifestations attributable to *Leishmania* infection were recorded. It also responded well to long-term allopurinol treatment with gradual disappearance of its dermal abnormalities and a considerable decrease in antibody levels over time, reaching a level that would be considered below the cut-off for dogs with *L. infantum* infection.

The mild neutrophilia found on the dog’s second follow-up at the HUSVM could be attributed to an inflammatory response to the dermatitis or to possible secondary bacterial infection of the skin. The mild hypoalbuminemia may also be explained by an inflammatory response as albumin is a negative acute phase protein which may decrease during inflammation [16]. The seropositivity to *L. infantum* antigen was not surprising as there is a strong serological cross-reactivity between different *Leishmania* species and it was also reported in cases of canine *L. tropica* infection [5, 17, 18]. The presence of *L. major* in regional lymph node, as found in this dog has also been demonstrated in human patients with this disease and indicates that the parasite may reach the lymph nodes draining the local cutaneous sites of infection [19]. The excellent response to allopurinol treatment and the healing of the dog’s lesions suggest that allopurinol used as the major drug against canine *L. infantum* infection [3], is also effective against canine *L. major* infection.

Very few studies have evaluated the involvement of companion animals in the epidemiology of *L. major* infection. A study from the Kerman province in southeast Iran, a focus of human *L. major* cutaneous leishmaniasis, has evaluated dogs and rodents as reservoirs for this infection and found no evidence of canine involvement [20]. Nevertheless, another study from Turkey detected widespread sub-clinical *L. major* and *L. tropica* infections in cats from the Aegean region of Turkey [21]. Further studies should be conducted in *L. major* foci to evaluate the possible involvement of domestic and wildlife carnivores in the epidemiology of this widespread zoonotic human infection.

## Conclusions

This is the first molecularly-confirmed report of clinical *L. major* infection in a dog and its response to anti-leishmanial treatment. Domestic and wild canine infection with *L. major* may be more prevalent in areas of endemic human *L. major* cutaneous leishmaniasis than currently recognized, and canines should be evaluated as possible additional reservoirs for human infection.

## Animal ethics statement

This study was carried out in accordance with the Hebrew University ethic regulations for experimentation in animals. The study involved exclusive use of samples taken as a part of the animal’s diagnostic procedure by attending veterinarians.

## Abbreviations

CBC: complete blood count
HRM: high resolutions melt
HUSVM: Hebrew University School of Veterinary Medicine
ITS: internal transcribed spacer
PCR: polymerase chain reaction
